# Toll-Like Receptor Mediated Modulation of T Cell Response by Commensal Intestinal Microbiota as a Trigger for Autoimmune Arthritis

**DOI:** 10.1155/2015/527696

**Published:** 2015-02-23

**Authors:** Rebecca Rogier, Marije I. Koenders, Shahla Abdollahi-Roodsaz

**Affiliations:** Experimental Rheumatology, Radboud University Medical Center, 6500 HB Nijmegen, Netherlands

## Abstract

In autoimmune diseases, a disturbance of the balance between T helper 17 (Th17) and regulatory T cells (Tregs) is often observed. This disturbed balance is also the case in rheumatoid arthritis (RA). Genetic predisposition to RA confers the presence of several polymorphisms mainly regulating activation of T lymphocytes. However, the presence of susceptibility factors is neither necessary nor sufficient to explain the disease development, emphasizing the importance of environmental factors. Multiple studies have shown that commensal gut microbiota is of great influence on immune homeostasis and can trigger the development of autoimmune diseases by favoring induction of Th17 cells over Tregs. However the mechanism by which intestinal microbiota influences the Th cell balance is not completely understood. Here we review the current evidence supporting the involvement of commensal intestinal microbiota in rheumatoid arthritis, along with a potential role of Toll-like receptors (TLRs) in modulating the relevant Th cell responses to trigger autoimmunity. A better understanding of TLR triggering by intestinal microbiota and subsequent T cell activation might offer new perspectives for manipulating the T cell response in RA patients and may lead to the discovery of new therapeutic targets or even preventive measures.

## 1. Introduction

Rheumatoid arthritis (RA) is a systemic autoimmune disease, which is characterized by chronic inflammation and progressive cartilage and bone destruction in multiple joints. A world-wide prevalence of about 1% ranks RA among the most-common autoimmune disorders [1]. Current therapy of RA is based on a choice, or often a combination, of nonsteroidal anti-inflammatory drugs (NSAIDs), disease-modifying antirheumatic drugs (DMARDs), glucocorticoids, and recently the so-called Biologicals targeting specific cytokines or certain immune cells.

The etiopathology of RA is complex, because cells of the innate and adaptive immune system as well as joint resident cells such as fibroblasts and chondrocytes contribute to the development and progression of RA [2]. The production of proinflammatory cytokines such as tumor necrosis factor (TNF) *α* and interleukin (IL)-1 and activation of lymphocytes are considered to play important roles in RA pathogenesis [3, 4]. A specific subset of T cells, known as T helper 17 (Th17) cells, is considered to be a major pathogenic mediator in RA [3, 5, 6].

Although the exact etiology remains unclear to date, RA is generally considered a multifactorial disease in which both genetic and environmental factors play a role [7]. Epidemiological studies have revealed that the largest genetic risk factors for RA are certain alleles of the HLA-DR gene [8]. In addition, polymorphisms in protein tyrosine phosphatase N22 (PTPN22), peptidyl arginine deiminase type IV (PADI4), signal transducer and activator of transcription 4 (STAT4), and TNF receptor-associated factor 1/complement C5 (TRAF1/C5) were found associated with RA [8]. However, the presence of susceptibility factors is neither necessary nor sufficient to explain the disease development, underlining a critical role for environmental factors.

Meta-analysis has shown that smoking is one of the environmental factors associated with RA pathogenesis [9]. In addition to smoking, periodontal pathogens such as* Porphyromonas gingivalis* and the induced periodontal disease have been implicated in the etiology of RA [10, 11]. Besides infectious bacteria, commensal bacteria have been implicated in the pathogenesis of RA [12]. In addition, there is strong evidence that Toll-like receptors (TLRs), which recognize microbial products, contribute to RA progression [13–15].

**Figure 1:**
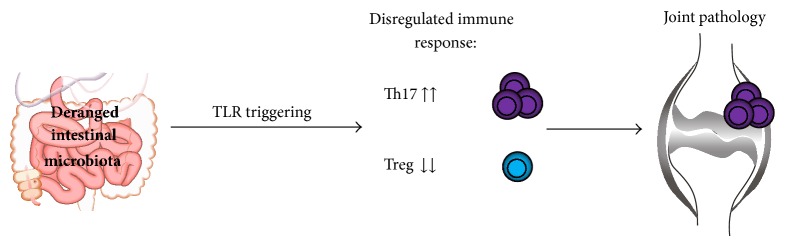
Exposure to deranged intestinal microbiota or a disregulated immune response to microbiota drives rheumatoid arthritis by promoting Th17 and deranging Treg cells.

Most of the polymorphisms associated with RA are involved in regulating T cell activation [16]. The genetically altered T cells are* potentially* autoreactive, that is, they may react to self-antigens in the joint and cause autoimmunity; nevertheless, the “naïve” T cells (called Th0) first need to become activated and acquire a pathogenic phenotype in order to be harmful. Exposure to (deranged) intestinal microbiota may be a critical factor. The aim of this review is to discuss the role of intestinal bacteria in the development of RA in the context of T cell modulation and the possible role that TLRs play in this process (Figure 1).

## 2. Th17 Cells and Rheumatoid Arthritis

Th17 cells protect against bacterial and fungal infections; however they also promote the development of autoimmune diseases such as multiple sclerosis, inflammatory bowel disease, psoriasis, and RA [17–22]. Regulatory T cells (Tregs) downregulate inflammation and serve to prevent tissue damage and autoimmunity. The balance between Th17 cells and Tregs is strictly regulated, and imbalance is thought to promote autoimmunity [23]. In RA, increased percentages of Th17 cells have been found in peripheral blood mononuclear cells (PBMCs) of patients [22]. These Th17 cells were shown to be potent inducers of matrix metalloproteinases and proinflammatory cytokines upon interaction with synovial fibroblast, thereby contributing to joint damage [22].

Other studies found increased levels of Th17 cells and decreased levels of Tregs in peripheral blood of patients with active RA [24, 25]. Furthermore, RA patients have Tregs with decreased suppressive activity [26]. Transforming growth factor (TGF) *β* is a key factor involved in maintaining the Th17/Treg cell balance: TGF*β* in combination with IL-6 or IL-21 promotes Th17 differentiation, but when TGF*β* is present in combination with IL-2, it will induce differentiation of Tregs [27, 28]. Inhibition of IL-6 function was shown to correct the Th17/Treg cell imbalance in RA patients [24]. Targeting the Th17 pathway in autoimmune diseases such as RA is very promising [29]. However, factors promoting Th17 differentiation are poorly understood. Since specific intestinal microbiota greatly promotes Th17 differentiation in intestinal mucosa, exposure to (deranged) intestinal microbiota may be a critical factor in autoimmune arthritis.

## 3. Intestinal Microbiota and Regulation of the Immune Response

Large numbers of commensal microorganisms inhabit the gastrointestinal tract of mammals. It has been shown that this commensal microbiota is essential for a proper development of the immune system, as GF mice possess an underdeveloped mucosal immune system [30]. GF mice have decreased numbers of lamina propria CD4^+^ cells, hypoplastic Peyer's patches, and greatly reduced immunoglobulin A (IgA) producing plasma cells [30, 31]. In addition, also spleen and lymph nodes are underdeveloped in GF mice, as they contain poorly formed B and T cell zones [30]. Introduction of* Bacteroides fragilis* into GF mice has been shown to induce correct development of the immune system [32].

Ivanov et al. showed that the introduction of SFB in GF mice resulted in an increase of Th17 cells in the intestinal lamina propria [33]. In the murine gut, the presence of SFB has been shown to promote the development of Th17-mediated autoimmune diseases such as experimental autoimmune encephalomyelitis (EAE), colitis, and arthritis [34–36]. Colonization of mice with* B. fragilis*, a human commensal, induces Tregs and prevents development of 2,4,6-trinitrobenzene sulfonic acid- (TNBS-) induced colitis [37]. In addition, oral treatment of mice with polysaccharide A (PSA), a molecule expressed by* B. fragilis*, induced IL-10 producing Tregs and protected against EAE [38]. Another study showed that colonization of mice with microbiota belonging to the* Clostridium* species also resulted in the induction of Tregs [39]. In addition, colonization of young mice with mix of* Clostridium* species resulted in resistance to dextran sodium sulfate- (DSS-) induced colitis [39]. These studies suggest that intestinal microbiota plays an important role in maintaining the balance between pro- and anti-inflammatory T cells, thereby preserving intestinal homeostasis.

A recent study elegantly demonstrated the specific labeling and tracking of intestinal leukocytes [40]. It was shown that intestinal leukocytes migrate to and from the intestine at steady state [40]. In addition, the migration of intestinal Th17 cells in arthritic K/BxN mice was studied and showed that gut derived Th17 cells end up in the spleen [40]. The fraction of gut-derived Th17 cells present in the spleen correlated with serum level of pathogenic auto antibodies [40]. This is the first study which shows that gut-derived Th17 cells can contribute to autoimmune arthritis [40].

Taken together, it is conceivable that a disturbed balance in the composition of microbiota, the so-called dysbiosis, could result in disruption of intestinal and systemic immune homeostasis. A link between intestinal microbiota and autoimmune deficiencies such as RA seems therefore plausible [41].

## 4. Rheumatoid Arthritis and Microbiota

Treatment with tetracycline antibiotics, in particular minocycline, was shown to significantly reduce disease activity in seropositive RA patients with disease duration of <1 year [42]. Moreover, the commonly used DMARD sulfasalazine is known to have both anti-inflammatory and antimicrobial properties [43]. Using a small set of oligonucleotide probes detecting broad groups of bacteria, intestinal microbiota of RA patients was found different from that of fibromyalgia (FM) patients [44]. The authors did not include healthy control subjects in the study; however a group of patients with FM, having a comparable age and sex distribution and receiving similar treatment with NSAIDS drugs, were included as controls. This study showed that RA patients had significantly less* bifidobacteria* species, bacteria of the* Bacteroides-Porphyromonas-Prevotella* group,* Bacteroides fragilis* subgroup, and the* Eubacterium rectal-Clostridium coccoides* group, when compared to FM patients [44].

A recent study using 454 pyrosequencing of 16S rRNA of intestinal microbiota in stool samples found a strong correlation between the presence of* Prevotella copri* with disease in new-onset untreated RA patients [45]. Abundance of* P. copri* in this study was inversely correlated with the presence of HLA-DRB-1 risk alleles, suggesting requirement of intestinal microbial signals in the absence of genetic predisposition factors for one to develop the disease. Another study demonstrated that fecal microbiota of RA patients contained significantly more* Lactobacilli* compared to healthy controls [46]. Altogether, the efficacy of oral antibiotic treatment and recent findings on disturbed composition of intestinal microbiota in early RA supports the involvement of microbiota in the development of RA.

## 5. Experimental Evidence on the Role of Commensal Microbiota in Arthritis

The critical role of commensal microbiota in the development of arthritis has been shown in at least three spontaneous autoimmune models of arthritis. These studies showed that spontaneous disease in mice with T cell-activating genetic modifications is greatly diminished under germ-free (GF) or specified pathogen-free (SPF) conditions [13, 36, 47]. Another study showed that oral treatment with enrofloxacin, a broad-spectrum antibiotic, exacerbates collagen induced arthritis (CIA) in mice by influencing production of a number of proinflammatory cytokines such as IL-6 and IL-17 [48].

IL-1 receptor antagonist (IL-1Ra) deficient mice spontaneously develop autoimmune arthritis due to excessive IL-1 signaling [49]. Development of autoimmune arthritis in this mouse model is dependent on microbial flora, as arthritis was strongly attenuated in GF IL-1Ra^−/−^ mice [13]. Colonization* with Lactobacillus bifidus* resulted in arthritis with incidence and severity scores comparable to those observed in conventionally housed mice [13]. The GF status IL-1Ra^−/−^ mice resulted in a notable decrease in IL-17 and IL-1*β* production by splenocytes upon CD3 as well as TLR2 and TLR4 stimulation, suggesting abolishment of Th17 differentiation [13].

SKG mice have a mutation in the gene encoding an SH2 domain of ZAP-70, a signal transduction molecule in T cells. The aberrant ZAP-70 is thought to change the thresholds of T cells to thymic selection, which results in the positive selection of otherwise negatively selected autoimmunity T cells [50]. SKG mice develop chronic autoimmune arthritis under conventional conditions; however in strictly controlled SPF environment arthritis failed to develop [47]. Arthritis in SKG mice was shown to be accompanied with high sera levels of IL-6, known to be important in Th17 induction. However, in sera from SKG mice kept in SPF conditions IL-6 was undetectable [47].

T cells of K/BxN mice express a transgenic T cell receptor which recognizes a self-peptide derive from glucose-6-phosphate isomerase (GPI). These autoreactive T cells stimulate GPI-specific B cells to produce high amounts of GPI autoantibodies. Th17 cells seem to be driving arthritis in this model, as neutralization of IL-17 blocked the development in SPF-housed K/BxN mice [36]. Intriguingly, GF K/BxN mice have an almost complete deficiency of Th17 cells and are protected from severe arthritis [36]. Moreover, oral treatment of K/BxN mice with vancomycin or ampicillin inhibited the development of arthritis, while in neomycin-treated mice disease was aggravated [36]. Introduction of segmented filamentous bacteria (SFB), a gut-residing bacteria, in GF K/BxN mice resulted in an increase of Th17 cells in the lamina propria and in onset of arthritis [36]. These results suggest that certain intestinal microbiota is able to trigger an imbalance in the T cell response which leads to the development of autoimmune arthritis in a genetically predisposed host.

## 6. TLR-Mediated Interactions between Bacterial Antigens and the Immune System

Although the mechanism by which commensal intestinal microbiota triggers the development of autoimmune diseases remains poorly understood to date, TLRs are most likely involved. TLRs recognize microbe-associated molecular patterns (MAMPs), which are shared by many microorganisms [51]. Each TLR recognizes certain MAMPs; for instance, TLR2, TLR4, TLR5, and TLR9 recognize peptidoglycans, lipopolysaccharides (LPS), flagellin, and unmethylated CpG motifs in bacterial DNA, respectively [52]. TLRs are expressed by a number of immune cells, including dendritic cells (DCs), macrophages, neutrophils, T cells, and B cells, but TLRs have also been found on resident cells, such as epithelial and endothelial cells [53].

**Figure 2:**
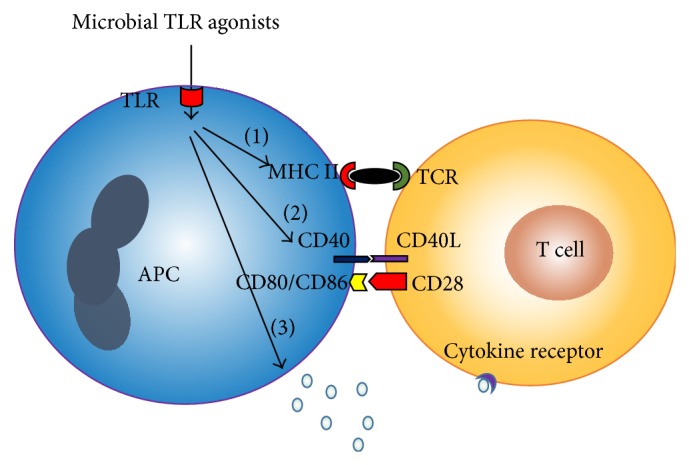
Toll-like receptor (TLR) activation on antigen presenting cells (APCs) enhances the antigenic signal to T cells. TLR activation induces the upregulation of MHC II (1), costimulatory molecules such as CD80, CD86, and CD40 (2), and release of cytokines (3).

Antigen presenting cells (APCs) such as DCs and macrophages are known to express TLRs, and activation of TLRs induces the upregulation of MHC class II molecules and thereby may substantially influence the strength of the antigenic signal to T cells in the “immunological synapse” [54] (Figure 2). Furthermore, activation of TLRs induces upregulation of costimulatory molecules such as CD80, CD86, and CD40, which provide the second signal for T cell activation (Figure 2). The third signal for T cell activation and differentiation, the cytokine environment, is dramatically affected by the type and the extent of TLR activation (Figure 2). For instance, activation of TLR4 and TLR9 is thought to skew T cell differentiation toward the Th1 phenotype through induction of IL-12 production by DCs, whereas TLR2 activation might induce a Th2-biased immune response through production of IL-10 and IL-13 [55–61]. TLR4-induced IL-23 contributes to the expansion and survival of Th17 cells [62]. In addition, conditioned medium from TLR4-stimulated DCs or PBMCs induces Th17 differentiation and IL-17 production, a process potentiated by TGF*β* [63].

In addition to the type of TLR activation, the extent of TLR triggering also seems to determine the type of immune response generated. For instance, it was demonstrated that a high dose of LPS triggers a Th1 response via TLR4 while a low LPS dose results in a Th2 response to an inhaled antigen [64]. Besides APC-mediated T cell activation, some TLRs such as TLR2, 5, and 7/8 are functionally expressed on T cells and directly cause T cell activation and proliferation upon stimulation [65–67]. Others (TLR3 and TLR9) can enhance survival of activated CD4^+^ T cells [68].

Also joint resident cells are known to functionally express TLRs. RA synovial fibroblasts (RASF) for instance are known to express TLR2, TLR3, TLR4, and TLR9 [69]. Stimulation of RASF with TLR2, TLR3, and TLR4 antigens (peptidoglycans, polyinosinic:polycytidylic acid, and LPS, resp.) results in high production of inflammatory cytokines, MMPs, and vascular endothelial growth factor and results in exacerbation of the Th1 and Th17 response [69].

A study with TLR deficient IL-1Ra^−/−^ mice demonstrated that TLRs play distinct roles in the regulation of the T cell balance. In this study it was shown that Th17 differentiation is reduced in TLR4 deficient IL-1Ra^−/−^ mice, while TLR2^−/−^ deficiency results in a shift in T cell balance from Th2 and Treg towards Th1 cells [13]. In addition, it was shown that IL-1Ra^−/−^ TLR2^−/−^ mice develop a more severe arthritis compared to IL-1Ra^−/−^ TLR2^+/+^ mice [13]. In contrast, TLR4 deficiency in IL-1Ra^−/−^ mice resulted in protection against severe arthritis [13]. This study shows that sensing of microbiota by TLRs plays a critical role in maintaining T cell balance and arthritis development.

## 7. Intestinal TLR Triggering

Commensal bacteria normally do not cross the epithelial barrier. A specific population of CX3CR1 expressing cells in lamina propria has been shown to sample the lumen and interact with commensal bacteria in the lumen [70]. Although, these cells were first identified as DCs, recent studies demonstrated that CX3CR1 expressing cells in the gut are more similar to macrophages than DCs [71, 72]. This is based on the observation that CX3CR1 expressing in the intestinal lamina propria are nonmigratory and cannot prime naïve T cells [71, 72]. However, another study identified CD103^−^ CD11b^+^ DCs which also express CX3CR1; these cells lacked macrophage markers such as F4/80 or CD64 [73]. CX3CR1 expressing cells were thought to be nonmigratory; however a recent study showed that these cells do migrate to mesenteric lymph nodes after antibiotic-induced dysbiosis and in the absence of MyD88 [74]. Despite this finding, it is believed that the CD11b^+^ CD103^+^ classical DC subset is mainly responsible for presentation of bacterial antigen to naïve CD4^+^ T cells and Th17 differentiation in the intestinal lamina propria [74–76]. Stimulation of CD11b^+^ CD103^+^ cells with flagellin, a TLR5 ligand, resulted in the expression of high amounts of IL-23 [76]. A recent study identified a subset of CCR2-expressing CD103^−^ CD11b^+^ DCs, in lamina propria which were able to drive IL-17 production in vitro [77]. These DCs produced IL-12 and IL-23p40, and production of these cytokines increased in response to TLR4 stimulation with LPS. These DCs were found in murine as well as human lamina propria [77].

A recent study showed that luminal bacteria stimulate the recruitment of CD103^+^ DCs to the epithelium, where these DCs can also sample the lumen [78]. Recruitment of the DC to the epithelium was shown to be depending on chemokines and TLR signaling [78]. Another study showed that TLR5 is highly expressed in DCs in the intestinal mucosa, but not in splenic DCs [79]. This same study showed that TLR5^−/−^ mice had increased Treg levels in the intestinal lamina propria, which suggests that TLR5 plays a role in regulating the intestinal Th17/Treg cell balance [79]. Another study demonstrated that TLR5 is expressed by CD11c^hi^  CD11b^hi^ DCs in lamina propria of mice [80]. These intestinal DCs induce the differentiation of Th1 and Th17 cells in response to flagellin [80]. In addition, TLR9 deficient mice were shown to have more Tregs and reduced Th1 and Th17 cell levels in the intestine [81].

Besides DCs also intestinal epithelial cells (IECs) in the gut are known to express TLRs. TLR 1, 2, 3, 4, 5, and 9 are known to be expressed by IECs in human small intestine, and TLR1-9 have been shown to be present on IEC in the colon [82]. In the mouse TLR1, 2, 3, 4, 5, 9, and 11 have been detected in the small intestine, and in the colon TLR2, 3, 4, and 9 were shown to be present [82]. The expression of TLRs in the gut seems to be regulated by commensal bacteria, as it was shown that the expression of TLR2, 3, 4, and 5 was higher in colonic epithelial cells of specific pathogen-free mice when compared to GF mice [83]. An in vitro study showed that TLR4 and basolateral TLR9 stimulation on IECs drives an inflammatory response [84]. However, apical TLR9 activation resulted in the production and secretion of galectin-9, which was shown to support the development of Tregs [85].

TLR signaling on IEC is also important in maintaining the epithelial barrier; for instance, TLR2 activation on epithelial cells protects against barrier disruption by upregulating the expression of zonula occludens, while TLR4 signaling results in increased intestinal permeability through upregulation of membrane protein kinase C activity [86, 87]. Translocation of bacteria across the membrane will result in an inflammatory response in the intestinal lamina propria. It has been hypothesized that intestinal barrier function, in particular the intercellular tight junctions modulated by zonulin among others, may be impaired in autoimmune disease [88, 89]. However, it is not yet clear whether this is indeed the case in individuals with autoimmune diseases such as RA.

As mentioned before a shift in the Th17/Treg cell balance is considered to be an important aspect of autoimmunity. The studies discussed here indicate an important role of intestinal TLR triggering in shaping the T helper cell subsets. This makes microbial recognition in the intestine interesting in the context of autoimmune diseases such as RA. The studies quoted here are mainly in mice. The role of intestinal TLR triggering in shaping the T cell response in humans remains mainly unclear and warrants thorough future investigation.

## 8. Specific Bacteria Shape the Intestinal Immune Response

Round et al. showed that polysaccharide A (PSA) of* B. fragilis* activated TLR2 directly on Tregs, which resulted in activation of these Tregs [90]. However,* B. fragilis *lacking PSA induces a Th17 response, which suggests that PSA induces an anti-inflammatory response through activation of TLR2 [90]. In addition, it was shown that PSA of* B. fragilis* prevents TNBS-induced colitis by inducing IL-10 producing Tregs. However, PSA induced protection was absent in TLR2^−/−^ mice indicating that TLR2 signaling is required for PSA-induced protection [37]. Another study showed that* B. fragilis* is able to release PSA in outer membrane vesicles which are sensed by DCs through TLR2 resulting in induction of Tregs and IL-10 production [91].

A recent study showed that presentation of SFB antigens by MHCII^+^ CD11c^+^ intestinal DCs is required for mucosal Th17 cell differentiation [92]. In MHCII deficient mice, no SFB-induced Th17 differentiation was observed; however recovery of MHCII expression on only CD11c^+^ cells was able to rescue Th17 induction [92]. In mice lacking peripheral lymph nodes and gut-associated lymphoid tissue, SFB induced Th17 priming was unaffected, suggesting that SFB-induced T cell priming takes place in the lamina propria [92]. It is likely that the presence of SFB also triggers TLR signaling. SFB encode four types of flagellin, three of which are recognized by TLR5 [93]. In the mouse gut TLR5 is expressed by CD11c^hi^  CD11b^hi^ DCs in lamina propria which induce the differentiation of Th1 and Th17 cells in response to flagellin [80]. This suggests that SFB skew T cell differentiation via TLR5 triggering. Involvement of TLRs in bacteria-induced mucosal T cell responses and the subsequent systemic autoimmunity seems therefore plausible.

## 9. Conclusion

Results of multiple studies show that commensal intestinal microbiota affect the Th17/Treg cell balance in the lamina propria and that intestinal Th17 cells can promote experimental arthritis [33, 36, 37, 39]. In addition, studies with experimental models of arthritis suggest that recognition of intestinal microbiota is required for the onset of autoimmune arthritis [13, 36, 47]. It is likely that TLRs mediate the effects of intestinal microbiota on Th cell differentiation in lamina propria. Multiple studies have shown that TLR activation plays an important role in shaping the intestinal T cell subsets [80, 84, 85, 90]. In addition, the study with IL-1Ra/TLR2 and IL-1Ra/TLR4 double gene deficient mice points toward an important role of these TLRs in T cell mediated autoimmune arthritis [13]. It remained unclear how microbiota-induced Th17 cells exactly contribute to systemic autoimmunity in RA. Cross-reactivity of bacteria-specific Th17 cells to endogenous (joint-derived) antigens is a possible mechanism. Another possibility is that microbiota induced T cells promote the differentiation of self-reactive Th17 cells by changing the cytokine environment. Migration of intestinal Th17 cells to the joint and subsequent production of proinflammatory mediators is another possible mechanism. A better understanding of these yet unexplored areas and the involvement of TLR triggering by intestinal microbiota in the gut in systemic autoimmunity might offer new perspectives for manipulating the T cell response in RA patients and may lead to the discovery of new therapeutic targets or even preventive measures.
